# Air-Compressor of the Testicle

**Published:** 1853-09

**Authors:** 


*Air-Compressor of the Testicle.* By Mr. HUTCHINSON. In the treatment of the various inflammatory enlargements of the testis, it is now generally admitted that few measures are so efficient as the application of pressure. As ordinarily effected, by means of strapping with adhesive plaster, it is, however, liable to several drawbacks. The adaptation of the straps is not easy, and, if awkwardly done, occasions great pain. In order to secure progress, it is necessary very frequently to change the plaster, for the casing ceases to be useful as soon as the enclosed gland has yielded to the compressing force. [illegible] removals and reäpplications of the dressings occasion great loss of time to the surgeon, and pain and inconvenience to the patient. To obviate these objections, an apparatus has been contrived by Mr. Hutchinson, which consists of a double bag (on the principle of the double nightcap) with the cavity of which a tube communicates. The whole is constructed of air-tight material, and to the end of the tube, which is long and flexible, a screw-cock is affixed. The enlarged testicle having been put into the involuted bag, the latter is kept in place by means of a ribbon tied tightly round the cord. Air is then blown through the tube into the cavity of the bag, and compression of the enclosed gland is effected, just as would be the case with the lung if air were forced into the pleural sac. When filled as full as the patient can comfortably bear, the screw-cock is closed, and the air retained. As soon as by diminution in the size of the testicle the tension has become much lessened a little more air may, without any necessity for changing the apparatus, be blown in, and thus a condition of constant and equable compression is sustained.—With regard to putting on the bag, no more difficulty is found in insulating the affected organ than is the case in applying strapping. The support afforded is very comfortable, and the inflamed part being protected on all sides from friction, blows, etc., by a cushion of air, the patient can walk about, or follow his usual avocations. It must, however, be admitted, that the bulk is considerably greater than that of a merely strapped gland. Its chief disadvantages, as far as it has yet been tried, appear to be that, from being made of non-permeable material, it entirely confines the cutaneous exhalations of the part, and also that the skin beneath the constricted neck is a little liable to excoriate. For the sake of cleanliness, therefore, the bag should be removed, and the skin washed once a day; if necessary, the latter may be smeared with oil, or protected by a layer of oiled silk. If the case be suitable, a piece of lint, smeared with mercurial ointment, may be wrapped round the testicle, and the bag applied over it. The cases in which Mr. Hutchinson has proposed to use his invention are chronic and subacute inflammation of the gland, varicocele, some peculiar forms of hydrocele, after paracentesis, and, possibly, malignant affections.—*Med. Times and Gaz.*

